# Implementing an evidence-based computerized decision support system to improve patient care in a general hospital: the CODES study protocol for a randomized controlled trial

**DOI:** 10.1186/s13012-016-0455-x

**Published:** 2016-07-07

**Authors:** Lorenzo Moja, Hernan Polo Friz, Matteo Capobussi, Koren Kwag, Rita Banzi, Francesca Ruggiero, Marien González-Lorenzo, Elisa Giulia Liberati, Massimo Mangia, Peter Nyberg, Ilkka Kunnamo, Claudio Cimminiello, Giuseppe Vighi, Jeremy Grimshaw, Stefanos Bonovas

**Affiliations:** 1 Department of Biomedical Sciences for Health, University of Milan, Via Pascal 36, 20133 Milan, Italy; 2 Clinical Epidemiology Unit, IRCCS Orthopedic Institute Galeazzi, Via Galeazzi 4, 20161 Milan, Italy; 3 Internal Medicine Division, Medical Department, Vimercate Hospital, Via Santi Cosma e Damiano 10, 20871 Vimercate, Italy; 4 School of Specialization in Hygiene and Preventive Medicine, University of Milan, Milan, Italy; 5 IRCCS Mario Negri Institute for Pharmacological Research, Via La Masa 19, 20156 Milan, Italy; 6 Department of Health Science, Centre for Medicine, University of Leicester, University Road, Leicester, LE1 7RH UK; 7 Medilogy Srl, Viale Monza 133, 20125 Milan, Italy; 8 Duodecim Medical Publications Ltd, Kaivokatu 10 A, 00101 Helsinki, Finland; 9 Ottawa Hospital Research Institute & Department of Medicine, University of Ottawa, 501 Smyth Road, Ottawa, ON K1H 8L6 Canada; 10 Humanitas Clinical and Research Center, Via Manzoni 56, 20089 Rozzano Milan, Italy

**Keywords:** Computerized decision support systems, Electronic health records, Evidence-based medicine, Pragmatic trial, Randomized controlled trial, Reminder systems

## Abstract

**Background:**

Computerized decision support systems (CDSSs) are information technology-based software that provide health professionals with actionable, patient-specific recommendations or guidelines for disease diagnosis, treatment, and management at the point-of-care. These messages are intelligently filtered to enhance the health and clinical care of patients. CDSSs may be integrated with patient electronic health records (EHRs) and evidence-based knowledge.

**Methods/design:**

We designed a pragmatic randomized controlled trial to evaluate the effectiveness of patient-specific, evidence-based reminders generated at the point-of-care by a multi-specialty decision support system on clinical practice and the quality of care. We will include all the patients admitted to the internal medicine department of one large general hospital. The primary outcome is the rate at which medical problems, which are detected by the decision support software and reported through the reminders, are resolved (i.e., resolution rates). Secondary outcomes are resolution rates for reminders specific to venous thromboembolism (VTE) prevention, in-hospital all causes and VTE-related mortality, and the length of hospital stay during the study period.

**Discussion:**

The adoption of CDSSs is likely to increase across healthcare systems due to growing concerns about the quality of medical care and discrepancy between real and ideal practice, continuous demands for a meaningful use of health information technology, and the increasing use of and familiarity with advanced technology among new generations of physicians. The results of our study will contribute to the current understanding of the effectiveness of CDSSs in primary care and hospital settings, thereby informing future research and healthcare policy questions related to the feasibility and value of CDSS use in healthcare systems. This trial is seconded by a specialty trial randomizing patients in an oncology setting (ONCO-CODES).

**Trial registration:**

ClinicalTrials.gov, https://clinicaltrials.gov/ct2/show/NCT02577198?term=NCT02577198&rank=1

## Background

### Background and rationale

Despite the proliferation of clinical guidelines and continued efforts by local and national healthcare systems to optimize decision-making on patient diagnosis, treatment, and management, the quality of medical care is variable and often suboptimal [1]. There remains an apparent discrepancy between the growing availability of scientific evidence and the application of this evidence into medical care [2, 3]. Non-adherence to evidence-based guidelines, medical errors, and omissions in everyday practice may occur because of time pressure, inexperience, reliance on memory, multitasking, and failures in healthcare team coordination.

Computerized decision support systems (CDSSs) are information technology-based software that provide health professionals with actionable, patient-specific recommendations or guidelines for clinical care at the point-of-care; these messages are intelligently filtered and presented at appropriate times during the decision-making process in order to enhance patients’ health [4, 5]. The opportunity to improve patient care by increasing clinicians’ accessibility to medical knowledge at the site of practice represents one of the main incentives for investing in the development and evaluation of these sophisticated information systems.

In particular, studies focusing on the effectiveness of “new generation” CDSSs demonstrate their potential to assist with problems raised in clinical practice, decrease the rate of medication errors, increase clinicians’ adherence to guideline- or protocol-based care, and, ultimately, improve the overall efficiency and quality of healthcare delivery systems [6–19]. These innovative systems can be integrated into hospital electronic health records (EHRs) and feature authoritative point-of-care information services and evidence-based knowledge [20].

This has led to some early work: a systematic review assessing the effectiveness of such new generation CDSSs demonstrated encouraging results [21]. Although this review did not show CDSSs to affect mortality, they were shown to moderately improve morbidity outcomes. Differences were further observed for costs and health services utilization, but these were often inconsistent in the direction of effect and small in magnitude. The conclusion of a landmark paper, published nearly 15 years ago, still reflects the current scenario: “Although the promise of clinical decision support system-facilitated evidence-based medicine is strong, substantial work remains to be done to realize the potential benefits” [22].

Current research on CDSSs suffers two noteworthy limitations [5]. First, while numerous studies have evaluated the effectiveness of CDSSs, comparatively few implemented a randomized controlled trial (RCT) design. Second, most published evaluations of the impact of CDSSs on healthcare quality were conducted in academic medical centers using “homegrown” systems that featured restricted clinical content for particular conditions (e.g., thromboprophylaxis). There is limited research on “mature” CDSSs that are commercially available and capable of supporting a wide range of clinical activities. Across countries, the adoption of these systems by hospitals is likely to increase in the future.

### Objective

Our study aims to evaluate the effectiveness of patient-specific, point-of-care reminders generated by the Medilogy Decision Support System (MediDSS) [23] on clinical practice and the quality of care in a general hospital.

## Methods/design

This protocol is reported in accordance with the SPIRIT 2013 guidance for content of clinical trial protocols [24, 25].

### Trial design

**Fig. 1:**
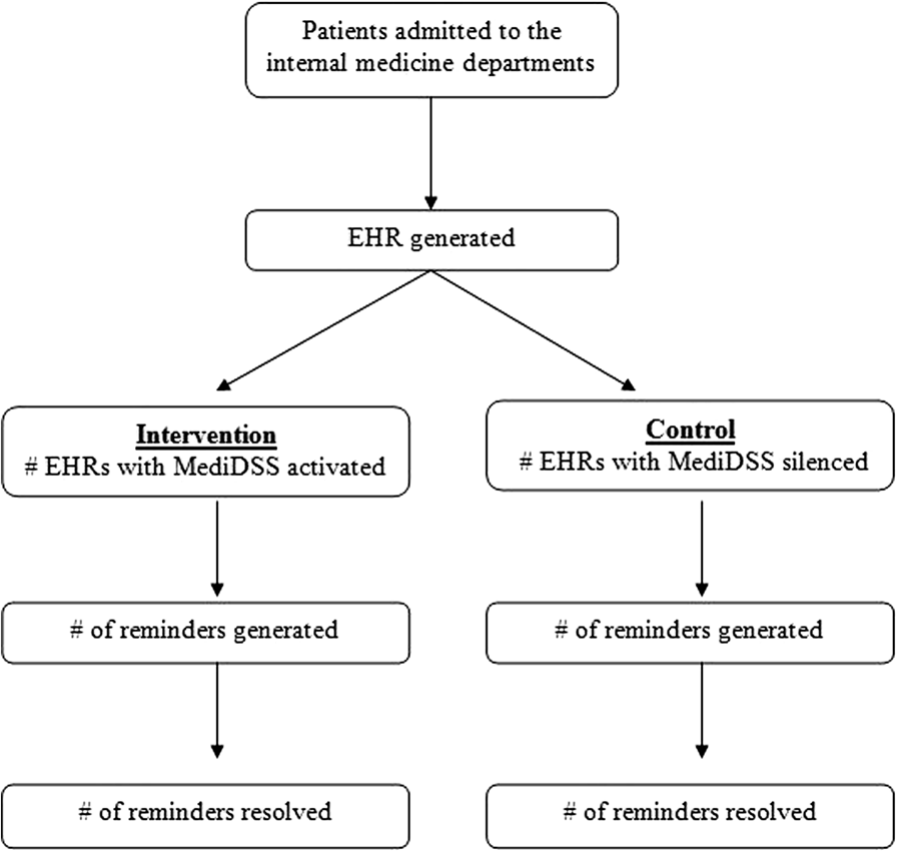
Trial flow chart

The CODES (computerized decision support) trial will implement a pragmatic, parallel group and randomized controlled design with a 1:1 allocation ratio. The flow diagram of the study can be found in Fig. 1.

### Study setting

The study will involve the medical staff of the internal medicine departments of the Vimercate Hospital from Azienda Ospedaliera di Desio e Vimercate (AODV) a multi-site hospital system located in the Lombardy region of Italy [26]. AODV includes hospitals and health units distributed in the Province of Monza and Brianza, which covers a population of approximately 850,000 inhabitants. The Vimercate Hospital has a medical staff of more than 230 doctors, a total of over 900 health professionals, and an overall capacity of 489 beds. It supports over 20 specialties and subspecialties. The catchment population is approximately 200,000 inhabitants, with more than 15,000 admissions per year. In addition to the in-patient wards, the hospital houses many facilities for diagnosis and treatment.

Since 2010, the Vimercate Hospital has been electronically tracking all clinical and administrative information through an EHR system based on the “Tabula Clinica” platform (developed by Dedalus S.p.A.) [27].

### Eligibility criteria and recruitment

As a pragmatic clinical trial [28, 29], CODES seeks to investigate the effectiveness of MediDSS reminders in everyday clinical practice with diverse patients and varying conditions. Thus, we will enroll all of the patients admitted into the internal medicine departments of the AODV, without applying any exclusion criteria.

### Intervention

We selected the MediDSS after a comparative assessment of available editorial products using a predefined set of essential criteria [30, 31]. MediDSS is a product by Medilogy, an Italian developer of scientific software and medical technology. Medilogy translated and adapted Evidence-Based Medicine electronic Decision Support (EBMeDS) [32], a CDSS developed by Duodecim Medical Publications Ltd., a company owned by the Finnish Medical Society Duodecim. EBMeDS can be described as a set of rules (scripts) based on EBM guidelines and applied to structured health data. MediDSS further includes knowledge from Swedish, Finnish, Interaction X-referencing (SFINX), a drug-drug interaction database containing concise evidence-based information about the harms and benefits of about 18,000 drug interactions and adverse events [33].

**Fig. 2:**
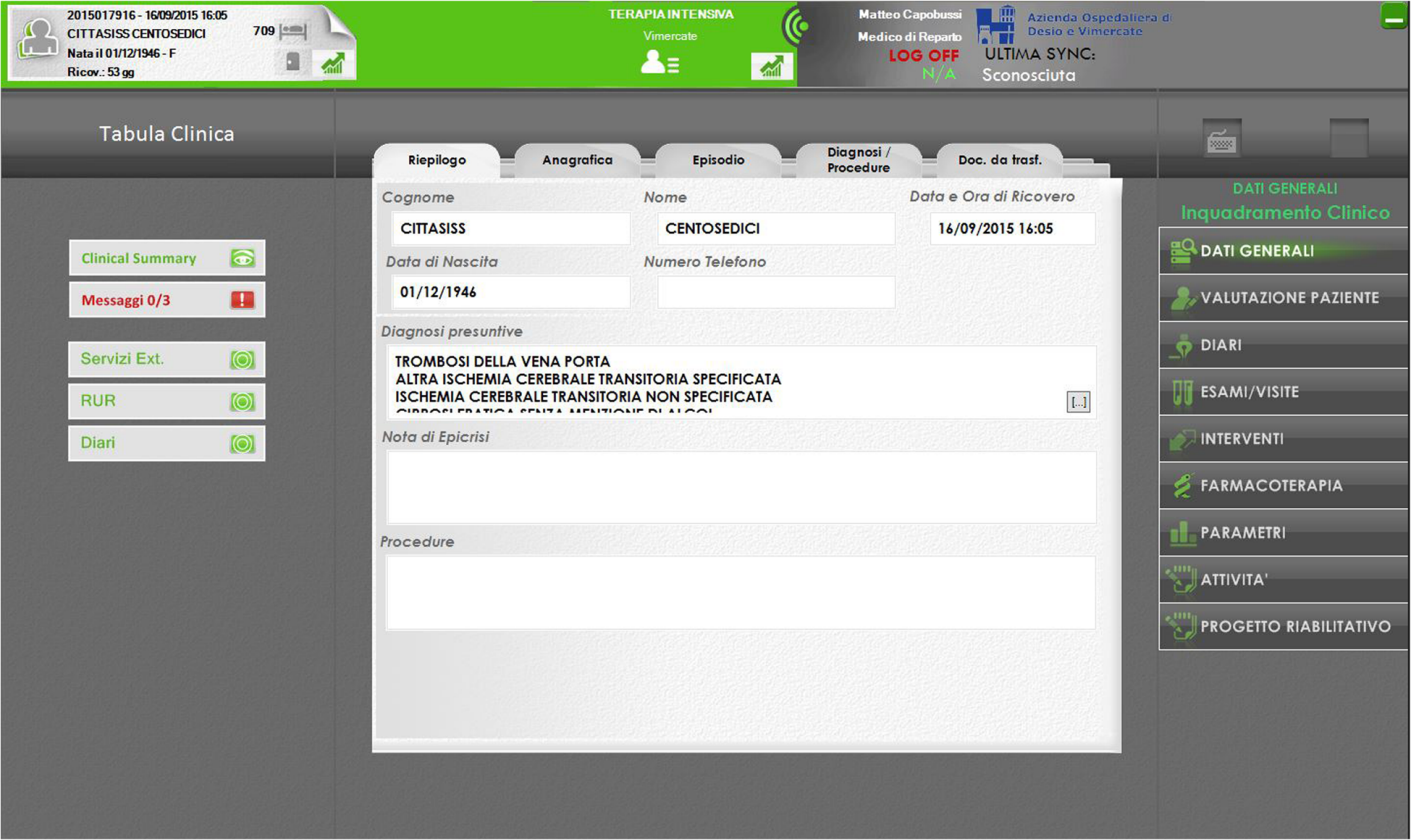
Screenshot of the CDSS activation button (in red)

**Fig. 3:**
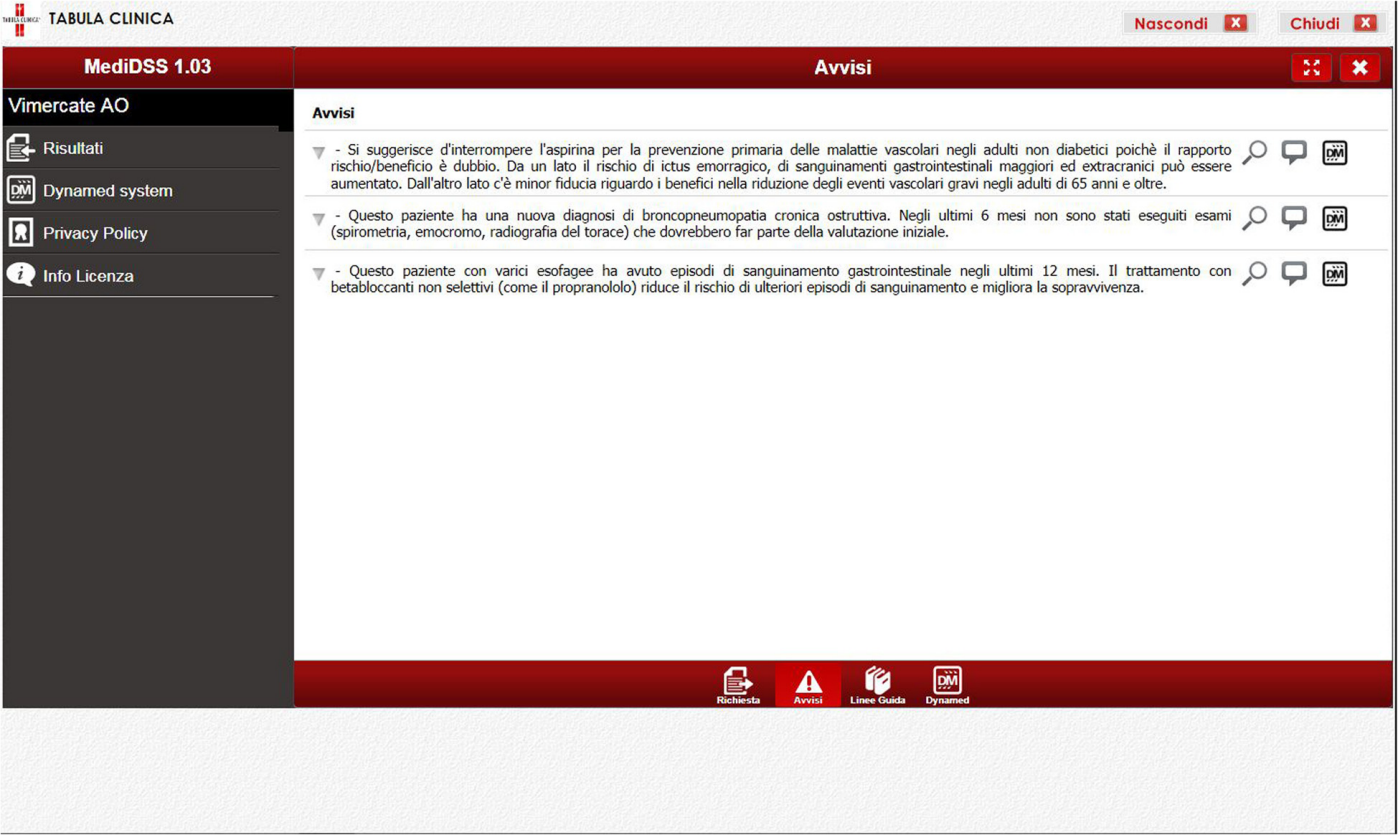
Screenshot of the CDSS activated online remiders

MediDSS may be used as a stand-alone application, or may integrate structured patient data from EHR to generate patient-specific reminders, therapeutic suggestions, and diagnosis-specific links to full-text guidelines. Reminders are automatically generated and displayed on the monitors of clinicians when they open a patient’s EHR, enter a new diagnosis, prescribe a drug, or when new laboratory test results are available. Reminders were formed using international evidence-based guidelines and subsequently approved by an international panel of experts. Our study will use international reminders (*n* = 262) that cover a large number of health conditions across specialties and are derived from the EBMeDS and SFINX database. In addition, 17 local reminders have been carefully selected by a team of doctors at Vimercate Hospital, along with members of the trial team. Table 1 reports some examples of the reminders. Figures 2 and 3 show a snapshot of the activation button and of the actual reminders.

**Table 1:** Examples of reminders generated by MediDSS from the EBMeDS and SFINX databases

Clinical reminders in MediDSS based on EBMeDS database	Drug-drug interaction reminders in MediDSS based on SFINX database [66]
Adjusting warfarin dose in atrial fibrillation: If a patient with atrial fibrillation and warfarin treatment, who has not had heart valve replacement, has not had an INR test during the last 8 weeks, the text “Last INR over 8 weeks ago, order INR?” is shown.	Interaction between spironolactone and potassium: “The combination of potassium supplements and potassium sparing diuretics can result in hyperkalemia.”
If a new INR result is outside the range 1.9–3.2, the text “Check warfarin dose (INR target 2.0–3.0, but note that if the patient has a mechanical mitral valve, the INR target is 2.5–3.5)” is shown, with link to dose calculator.	Interaction between warfarin and acetylsalicylic acid: “Concomitant use is associated with an increased risk of bleeding.”

MediDSS reminders will be shown on the EHR of patients only within the intervention group. During the care of control group patients, the generated reminders will not be shown to the physicians, such that the control is usual clinical practice without the use of the MediDSS service. However, physicians in both groups will have access to the best evidence for usual care at all times during the trial through the active searching of full-text EBM guidelines on the Internet. All participating physicians will be informed on the availability and use of the MediDSS system.

### Stepped wedge implementation

The intervention is a new technology: its integration in the current hospital system requires the configuration and customization of the software. To allow security controls and successful implementation, the CDSS will be sequentially rolled out to participants over a number of time periods. We anticipate that the number of periods will be limited (i.e., two or three periods). Over an initial period, all participants will receive the intervention. The order in which participants will receive the intervention is not determined at random, but will be determined by selecting physicians prone to provide constructive feedback to the implementation team. The RCT adopts a stepped wedge implementation of the intervention, but not a stepped wedge design [34]. Sequential roll-out of the intervention will not be considered a pilot phase of the trial, but a part of the whole RCT.

### Selection and development of priority reminders

In order to encourage the participation of the hospital staff within the study, we invited hospital representatives to assess the priority needs of the hospital wards and develop a set of reminders to address them. One topic of particular interest to the hospitals involved venous thromboembolism (VTE) prevention. The rationale for the prioritization of this condition is provided below:

(i) Despite evidence supporting the benefits of VTE prophylaxis based on the risk stratification process [35] as well as the availability of local hospital guidelines, the prophylactic drugs were inconsistently administered among patients.
(ii) The Vimercate Hospital has an increasingly large population of elderly (aged >65 years) and very elderly (aged >80 years) subjects, who have a higher risk of recurrent VTE and acute pulmonary embolism [36].
(iii) Between 2010 and 2012, 45 patients (9.6 %, 95 % CI 7.2–12.6) and 75 patients (16.0 %, 95 % CI 12.9–19.5) died, respectively, within 30 and 90 days after discharge due to VTE [36].
(iv) Research has shown the use of CDSSs to improve the assessment of patients’ risk for VTE, facilitate appropriate administration of prophylaxis interventions, and reduce the rate of symptomatic VTE in hospitalized patients [37–53].

In order to develop the set of reminders for VTE prevention, the local hospital expert group proposed a risk stratification process based on the Padua score [54]. This formula calculates the overall risk (low or high) of VTE for each patient using both clinical and surgical risk factors. Details of the underlying algorithm are provided in Table 2.

**Table 2:** Description of algorithm for the use of venous thromboembolism prevention therapy

I. The algorithm incorporates the Padua score [54], which uses ten common risk factors to identify patients at a high risk for VTE. Each risk factor is individually weighted according to a point-based scale. – Active cancer (defined as presence of metastases or recent chemotherapy), known thrombophilic condition, and reduced patient mobility are each assigned a score of 3 points. – Recent major surgery is assigned a score of 2 points. – Advanced age (greater than 70 years), obesity (BMI greater than 30), bed rest, and hormone replacement therapy or oral contraceptives are each assigned a score of 1 point. Patients are identified as a high risk for VTE if they accumulate a sum of 4 or more points. When the risk level is low, no medication is recommended; when the risk level is high, a prophylactic strategy using a high-dosage low-molecular-weight heparin is recommended. II. The second part of the algorithm involved the exclusion criteria for the use of VTE prophylaxis. – Home anticoagulant therapy – Contraindications to pharmacologic prophylaxis – Active bleeding	

Besides the VTE prevention therapy reminders, other scripts were chosen for development and tailored to the hospital’s needs. The following alerts were identified by the hospital’s clinicians as of special interest:

- Adjusting warfarin dose in atrial fibrillation
- Alert on heparin-induced thrombocytopenia
- Clopidogrel, prasugrel or ticagrelor, and aspirin in acute ST-segment elevation myocardial infarction (STEMI)
- Low-molecular-weight heparin as anticoagulant for patients with VTE and cancer
- Selection of antithrombotic therapy in atrial fibrillation on the basis of the CHA2DS2VASc score
- Supplementary laboratory measurements in warfarin therapy
- Low-dose aspirin: dosing in renal insufficiency
- Drug-drug interaction: aspirin and ACE inhibitor
- Warfarin and paracetamol: drug-drug interaction
- ACE inhibitor or sartan for diabetic patients with albuminuria
- Glimepiride warning in renal insufficiency
- LDL-cholesterol concentration in patients with type 2 diabetes
- High BNP or proBNP; untreated congestive heart failure (CHF)?
- Initial laboratory examinations in patients with congestive heart failure (CHF)
- Beta blockers in the prevention of gastrointestinal bleeding in patients with cirrhosis
- ACE inhibitors or angiotensin receptor blockers for patients with diabetes and hypertension but no microalbuminuria

### Qualitative integration

The validity of this RCT relies on the actual implementation of MediDSS by physicians in their clinical activities. Healthcare service studies on CDSSs, however, consistently suggest that the mere provision of such technology does not guarantee its uptake. In fact, even if a CDSS is readily available within a hospital, clinicians often fail to follow its recommendations, ignoring in some cases up to 96 % of its alerts [55]. Given this context, our RCT is informed by qualitative interviews aimed to detect the barriers and facilitators to MediDSS uptake as perceived by diverse health professionals involved in patient care (e.g., physicians and nurses). The interviews are a part of a larger cross-sectional study, which involves three Italian hospitals [56]. The interviews will explore variables that may hinder the use of a CDSS in everyday clinical practice, including technical (e.g., poor usability or knowledge of system), individual (e.g., negative perception of CDSS or EBM, lack of motivation), group or organizational (e.g., structural or administrative constraints), and cultural factors (e.g., adverse social norms).

When feasible, the trial will be tailored to address the specific needs emerging from the qualitative assessment. We will collect feedbacks about usability, possible errors, or inaccuracies of the information and recommendations provided. We will offer the best possible solutions to clinicians and hospital staff to overcome these problems. We will further organize and facilitate group discussions among participants to address negative perceptions or misleading beliefs about CDSSs. The qualitative study seeks to support the use of CDSS by participants, thus increasing the integrity of the intervention and associated compliance.

### Study outcomes

Primary outcome: the rate at which the medical problems, which are detected by the MediDSS software and reported through the reminders, are resolved (i.e., resolution rates).

Secondary outcomes: (i) resolution rates for the VTE prevention reminders, (ii) in-hospital all causes mortality (iii) VTE-related mortality, (iv) in-hospital morbidity for VTE-related causes, and (v) the length of hospital stay during the study period.

### Sample size

We calculated the sample size on the basis of the primary outcome. A previous systematic review assessing the effects of computer reminders delivered to clinicians at the point-of-care on healthcare processes and outcomes found a median improvement of 4.2 % in process adherence across all reported process outcomes [57]. Accordingly, assuming resolution rates of 5 % in the intervention group versus 3 % in the control group due to a possible group contamination, we calculated that a sample of 4230 reminders will be necessary to detect the difference between the two groups (power = 0.90; α = 0.05, two-sided; 1:1 allocation). Because estimates for intracluster correlation are not available, we increased the required sample size (by 10 %) to 4650 reminders to account for clustering by patient.

Moreover, based on a prior study evaluating EBMeDS, which recorded an average of 0.30 reminders per individuals triggered at baseline [58], we determined that a total number of 15,500 patients (7750 per group) need to be enrolled. This figure corresponds to a conservative estimate of the recruitment period of 24 months for the internal medicine departments of the Vimercate Hospital.

### Allocation and blinding

Anonymous patient identification (ID) numbers in the EHR system will be the unit of randomization. An individual external to the study group will generate the anonymous IDs using a formula based on patients’ unique fiscal code numbers.

We will randomly assign patients to either the control or experimental group with a 1:1 allocation. We will follow a computer-generated randomization schedule stratified by gender and age (0–30, 31–60, 61–80, >80 years) using permuted blocks of random sizes [59]. Patients will be randomized immediately after the first launch of their EHR (entry of demographic data by physicians at hospital admission), and the allocation will be maintained through successive admissions.

Patients and study investigators (i.e., researchers, statisticians, information technology specialists, and hospital representatives) will be blinded to the allocation of participants. We will maintain the blinding up to the dataset disclosure. On the other hand, blinding of physicians is not feasible due to the nature of the intervention: the physician will know that a patient has been allocated to the intervention group if an automatic, patient-specific reminder is displayed on the screen.

### Data collection

The data collection for this study will follow the standard data collection procedures of the AODV. We will collect demographic (i.e., gender, age) and administrative (i.e., anonymous patient ID, admission and discharge dates, diagnoses) data from the EHR archive on a daily basis. Information on reminders, including all scripts that have been activated in a patient’s record, will also be collected daily, but during the night, so as not to disturb or slow down the use of the patient EHR.

### Statistical methods

For the primary outcome (i.e., resolution rates), the reminder will serve as the unit of analysis, and the patient the clustering factor. The patient will be the unit of analysis for the secondary outcomes (i.e., length of stay and in-hospital mortality). All analyses will follow the intention-to-treat principle: patients will be analyzed in the group to which they have been randomized. Descriptive statistics will be presented as means ± standard deviations (SD), medians and interquartile ranges (IQR), or percentages when appropriate. We will compare continuous variables using the Student’s *t* test when normally distributed, and the non-parametric two-sample Wilcoxon rank-sum (Mann-Whitney) test when they are not normally distributed. We will compare categorical variables using the chi-squared test or the Fisher’s exact test, as appropriate. To model the resolution rates of the reminders, we will run a random effects logistic regression analysis, accounting for clustering by patient [60].

For hypothesis testing, we will consider a probability level of less than 0.05 as statistically significant. All statistical tests will be two-sided. We will use the Stata software to perform all statistical analyses (Stata Corp., College Station, TX, USA).

### Data monitoring

Data monitoring will inform the CODES trial conduct, identifying the potential need for adjustments:

(i) *Sample size recalculation*: Because the sample size calculation utilizes several assumptions, we will analyze the first batch of data collected and adjust the estimated sample size, if necessary, at the end of the sequential roll-out of the intervention. The 24-month recruitment period may also be adjusted, accordingly.
(ii) *Interim analysis*: We will perform an interim analysis on the primary endpoint after 50 % of the patients have been randomized, after 50 % of the expected events have occurred, or after 12 months of the study’s initiation (the assumed half-life of the trial), whichever occurs first. An independent statistician that is blind to the patient allocation will perform the analysis. This analysis will inform whether the intervention has been proven for efficacy (beyond reasonable doubt). We will subsequently decide whether (or not) it is necessary to modify the study or prematurely terminate it, if necessary.
(iii) *End of trial*: The end of trial will occur 30 days after the randomization of the last EHR. We will submit an end of trial notification and final report to the competent ethical committee, the AODV, and to the sponsor.

### Harms

We do not anticipate any harms (or other unintended effects) to study participants. Intervention and control groups will differ in the presence (intervention) or absence (control) of automatic reminders displayed on physicians’ monitors. Patients assigned to the control group will receive usual care without the reminders. Nevertheless, we will consult an external advisory board in the event that the discontinuation of the study becomes an option due to unforeseeable reasons.

### Ethical and regulatory considerations

This study is conducted in accordance with the principles of the Declaration of Helsinki (October 2013) [61]. As the CODES trial has a cluster design (several reminders, the unit of analysis, may derive from the same EHR, the unit of randomization), we followed the Ottawa statement to identify research participants and apply ethical and regulatory protections [62, 63]. The intervention (electronic CDSS reminders) does not directly target patients but physicians who can be considered as the participants of the study. The risks associated with the participation of physicians in the CODES trial are negligible. Physicians will be fully informed about the involvement of the AODV in the CODES trial and trained to use the intervention. Requiring the signed consent of each physician is not feasible and will impact on the validity and generalizability of study results. Some have argued that healthcare professionals have an obligation to participate in health system or knowledge translation research [64, 65]. We consider that the waiver of signed consents will not adversely affect the rights or welfare of the research participants.

### Protocol amendments

Any changes to the research protocol that may impact the study conduct (e.g., changes in study design, eligibility criteria, study outcomes, sample size, study procedures, or significant administrative aspects) will require a formal amendment of the protocol. We will communicate any such amendments to the trial registry (ClinicalTrials.gov) and notify the health authorities in accordance with the Italian regulations. We will further seek the approval of the Ethical Committee for any amendments to the protocol.

### Confidentiality

The trial staff will ensure the maintenance of participants’ anonymity. The participants will be identified only by their initials and anonymous patient ID number. Depersonalized data will be extracted from the EHR. All documents will be stored securely and accessible only by the trial investigators and authorized personnel.

Clinical data collected during the study will only be accessible to the staff at AODV, thus complying with the current medical practice of the hospitals. The trial investigators external to the hospitals (statistician, data manager, information technology personnel, etc.) will not have access to any information at the patients’ level.

The CODES trial will comply with the Italian Data Protection Act, which requires data to be anonymized as soon as it is practical to do so.

### Dissemination policy

The trial results will be posted on ClinicalTrials.gov as well as published in an open-access medical journal.

We will further disseminate the study results to the health professionals of AODV who are involved in the study.

## Discussion

### Strengths and limitations

The CODES trial has several strengths. First, the randomized controlled study design is recognized as the “gold standard” for testing intervention outcome hypotheses, allowing us to maximize the likelihood that the differences observed between groups are due to the intervention rather than potential confounding factors. Second, the pragmatic design of the study under conditions that mimics the actual use of CDSSs in practice increases the generalizability of the results as well as allows a more accurate estimation of the intervention’s true effectiveness. Third, the choice of an intention-to-treat analysis helps to ensure the pragmatic design of the study; in other words, although not all physicians may adhere to the reminders within the study, we anticipate that the lack of compliance with evidence-based recommendations occurs in everyday practice.

We must note the methodological limitation that physicians will not be blinded to the treatment allocation. When a patient-specific reminder is automatically displayed on the monitors, the physician will know that the particular patient belongs to the intervention group. We are aware that the unit of allocation (i.e., patient) and the lack of physician blinding can lead to possible group contamination as one physician can have both intervention and control group patients; in this case, a physician may apply the knowledge from a reminder generated for an intervention group patient to a control group patient. This possible learning effect (contamination of knowledge) can decrease the trial effect and lead to a more conservative effect estimate (i.e., towards the null). Randomization at the physician level, however, does not eliminate the possibility of contamination as physicians can care for patients across different wards; this level of randomization would, moreover, increase the organizational complexity of the study.

### Conclusion

The use of CDSSs in healthcare systems is likely to increase in the near future due to (i) growing concerns about the quality of medical care; (ii) continuous calls for a meaningful use of health information technology; and (iii) increasing use of and familiarity with advanced technology among new generations of physicians.

Through our pragmatic trial, we will contribute to the current research and understanding of the effectiveness of CDSSs in primary care and hospital settings. The results of our study may inform future research and healthcare policy questions related to the feasibility and value of CDSS use in healthcare systems.

### Trial status

The implementation phase of the study was completed in November 2015, when the CDSS (MediDSS) was fully integrated with the hospital’s EHR (Tabula Clinica). Subject recruitment and data collection began in December 2015 in the Vimercate Hospital.

## Abbreviations

CDSS, computerized decision support system; CODES, computerized decision support; EBM, evidence-based medicine; EBMeDS, Evidence-Based Medicine electronic Decision Support; EHR, electronic health record; GFR, glomerular filtration rate; IQR, interquartile range; MediDSS, Medilogy Decision Support System; RCT, randomized controlled trial; SD, standard deviation; VTE, venous thromboembolism
